# Genome-wide expression changes induced by bisphenol A, F and S in human stem cell derived hepatocyte-like cells

**DOI:** 10.17179/excli2020-2934

**Published:** 2020-11-04

**Authors:** B. Lucendo-Villarin, P. Nell, B. Hellwig, P. Filis, D. Feuerborn, P.J. O'Shaughnessy, P. Godoy, J. Rahnenführer, J.G. Hengstler, A. Cherianidou, A. Sachinidis, P.A. Fowler, D.C. Hay

**Affiliations:** 1 Centre for Regenerative Medicine, University of Edinburgh, Edinburgh, UK; 2 IfADo-Leibniz Research Centre for Working Environment and Human Factors at the Technical University Dortmund, Dortmund, Germany; 3 Department of Statistics, Technical University Dortmund, Dortmund, Germany; 4 Institute of Medical Sciences, University of Aberdeen, Aberdeen, UK; 5 Institute of Biodiversity, Animal Health & Comparative Medicine, University of Glasgow, UK; 6 Institute of Neurophysiology and Center for Molecular Medicine Cologne (CMMC), University of Cologne (UKK), Cologne, Germany

**Keywords:** bisphenol A, bisphenol F, bisphenol S, pluripotent stem cell, liver, hepatocyte-like cells

## Abstract

The debate about possible adverse effects of bisphenol A (BPA) has been ongoing for decades. Bisphenol F (BPF) and S (BPS) have been suggested as “safer” alternatives. In the present study we used hepatocyte-like cells (HLCs) derived from the human embryonic stem cell lines Man12 and H9 to compare the three bisphenol derivatives. Stem cell-derived progenitors were produced using an established system and were exposed to BPA, BPF and BPS for 8 days during their transition to HLCs. Subsequently, we examined cell viability, inhibition of cytochrome P450 (CYP) activity, and genome-wide RNA profiles. Sub-cytotoxic, inhibitory concentrations (IC_50_) of CYP3A were 20, 9.5 and 25 µM for BPA, BPF and BPS in Man12 derived HLCs, respectively. The corresponding concentrations for H9-derived HLCs were 19, 29 and 31 µM. These IC_50_ concentrations were used to study global expression changes in this *in vitro* study and are higher than unconjugated BPA in serum of the general population. A large overlap of up- as well as downregulated genes induced by the three bisphenol derivatives was seen. This is at least 28-fold higher compared to randomly expected gene expression changes. Moreover, highly significant correlations of expression changes induced by the three bisphenol derivatives were obtained in pairwise comparisons. Dysregulated genes were associated with reduced metabolic function, cellular differentiation, embryonic development, cell survival and apoptosis. In conclusion, no major differences in cytochrome inhibitory activities of BPA, BPF and BPS were observed and gene expression changes showed a high degree of similarity.

## Introduction

Debate concerning the possible adverse effects of bisphenol A (BPA) in humans, related primarily to its estrogenic activity, has been ongoing for more than 20 years (Vandenberg et al., 2010[47]; Hengstler et al., 2011[16]; Dietrich and Hengstler, 2016[8]). BPA is used in the production of polycarbonates and epoxy resins, a common constituent of modern day plastics (Vandenberg et al., 2010[47]; Rubin, 2011[36]). It was first synthesized in 1891 and industrial production began in the 1950s when the first epoxy resins were developed. It has been estimated that bisphenol A (BPA) production will reach ~7.3 million tonnes in 2023 (WMStrategy, 2019[51]) which represents a modest 3 % increase per year, over the period 2017-2023. 

BPA is well absorbed after oral administration (Hengstler et al., 2011[16]). In the liver it is metabolized to its glucuronide and sulfate conjugates followed by rapid urinary excretion. The half-life of BPA in humans has been calculated to range between 0.7 and 2.3 hours (Hengstler et al., 2011[16]). More recently, bisphenol F (BPF) and S (BPS) have been suggested as “safer” alternatives to BPA. However, these structurally similar compounds also induce estrogenic effects (Stroheker et al., 2003[41]; Higashihara et al., 2007[17]; Ullah et al., 2019[46]). In the present study, we compared BPA, BPF and BPS in genome-wide analyses, using stem cell-derived hepatocyte like cells (HLCs) that were generated by an established platform (Wang et al., 2017[49], 2019[50]). 

We report that the three bisphenols induce largely overlapping gene expression alterations at sub-cytotoxic concentrations in HLCs. The reduction of cytochrome P450 activity is also broadly similar when HLCs from both cell lines are exposed to the three bisphenols. Taken together, our stem cell-based model provides a comprehensive overview of human bisphenol exposure in the developing liver.

## Methods

### Cell culture and differentiation

The human pluripotent stem cell lines H9 (female) and Man12 (male) were cultured and differentiated as previously described (Wang et al., 2017[49]). Maintenance of hESC was performed on pre-coated laminin 521 (Biolamina) in serum-free medium mTeSR1 (STEMCELL Technologies) and maintained in a humidified 37 °C, 5 % CO_2_ incubator. Differentiation was initiated at 40 % confluence by replacing serum-free medium with endoderm differentiation medium: RPMI 1640 containing 1× B27 (Life Technologies), 100 ng/mL Activin A (R&D Systems), and 50 ng/mL Wnt3a (R&D Systems). The medium was changed every 24 hrs for 72 hrs. On day 3, endoderm differentiation medium was replaced with hepatoblast differentiation medium, and this was renewed every second day for a further 5 days. The medium consisted of knockout (KO)-DMEM, Serum replacement, 0.5 % Glutamax, 1 % non-essential amino acids, 0.2 % β-mercaptoethanol (all Life Technologies), and 1 % DMSO (Sigma). On day 8, differentiating cells were cultured in the hepatocyte maturation medium HepatoZYME (Life Technologies) containing 1 % Glutamax (Life Technologies), supplemented with 10 µM hydrocortisone (Sigma-Aldrich), 10 ng/ml hepatocyte growth factor (PeproTech) and 20 ng/ml oncostatin M (PeproTech). On day 9, the maturation media was replaced every 48 hrs with fresh media supplemented with vehicle (0.1 % DMSO) or bisphenol A, F or S (Sigma-Aldrich) for a further 8 days.

### Immunofluorescence

Cell cultures were fixed in 100 % ice-cold methanol at -20 °C for 30 min. Subsequently, fixed cells were washed twice with PBS at room temperature. Cell monolayers were blocked with PBS-0.1 % Tween containing 10 % BSA for 1 hr, and the monolayers were incubated with primary antibodies diluted in PBS-0.1 % Tween/1 % BSA at 4°C overnight (Supplementary Table 11). The following day, the primary antibody was removed, and the fixed monolayers were washed three times with PBS-0.1 % Tween/1 % BSA. Following this, the cells were incubated with the appropriate secondary antibody diluted in PBS/0.1 % Tween/1 % BSA for 1 hr at room temperature and washed three times with PBS. Cultures were then mounted with PermaFluor aqueous mounting medium (Thermo Scientific) and counterstained with NucBlue Hoechst 33342 (Sigma-Aldrich). The cells were imaged with an Axio Observer Z1 microscope with LD PlanNeoFluar objective lenses (Carl Zeiss). This microscope was coupled to a Zeiss AxioCamMR3 camera used for image acquisition. The images were processed through Zeiss Axiovision SE 64 Rel 4.8, with Zeiss Axiovision version 4.9.1.0 used to analyze the images. The percentage of positive cells and SD was calculated from eight fields of view.

### Cytochrome P450 assays

Cytochrome P450 3A and 1A2 activities were measured in HLCs at day 18 using pGlo technology according to the manufacturer's instructions (Promega) and specific units of activity were expressed as either relative light units (RLUs) per millilitre of medium per milligram of protein (BCA assay, Pierce), or as a percentage of Cytochrome P450 activity compared to the vehicle control. Model fidelity was ensured using multi-parametric analysis with 5 replicates performed in each experiment. The IC_50 _for each bisphenol was estimated from the function **f**(**x**) = **ax** + **b**.

### ATP synthesis and caspase activation assays

ATP synthesis and Caspase 3/7 activity were studied in HLCs on day 18 employing pGlo technology (Promega) as previously described (Szkolnicka et al., 2014[42]; Lucendo-Villarin et al., 2017[27]). The experiments are representative of 5 replicates. The IC_50_ for each bisphenol was estimated from the function **f**(**x**) = **ax** + **b**.

### Gene array analysis and bioinformatics

Gene array analysis was performed as described (Godoy et al., 2015[12]). Notably, we could not find any significant expression differences between vehicle controls and untreated controls in a pre-analysis. Normalization of the raw microarray data was done using the Frozen robust multiarray analysis (fRMA) algorithm (McCall et al., 2010[30]). To determine differentially expressed genes the R package limma was used (Smyth et al., 2005[40]). A separate model was fitted for each cell line to model the effect of the three bisphenols compared to the untreated control. Adjustment for multiple testing was conducted with the method of Benjamini and Hochberg (FDR, false discovery rate) (Benjamini and Hochberg 1995[4]). Genes with a fold chance >1.5 (upregulated) or <1/1.5 (downregulated), and an adjusted p-value <0.05 were considered differentially expressed. The overlap ratio can be used to quantify to which degree genes in an overlap are overrepresented. For pairwise overlap ratios the score is calculated as


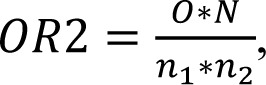


where *N* represents the total number of genes on the array, *n*_1_ the number of differentially expressed genes under condition 1, *n*_2_ the number of differentially expressed genes under condition 2, and *O* the number of genes in the overlap. A value of 1.0 indicates a random overlap, and values higher than 1.0 are indicative of an overlap that is higher than expected by chance in case of independence. The overlap ratio for the overlap of all three conditions is calculated as


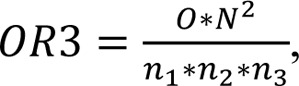


where *n*_3_ represents the number of differentially expressed genes under condition 3. Analogously to the case with two groups, the overlap ratio for three groups is the ratio of the proportion of genes in the overlap (*O*/*N*), compared to the expected value if the three groups were selected randomly and independently (*n*_1_/*N* * *n*_2_/*N* * *n*_3_/*N*), which results in


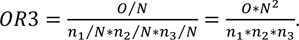


Equivalently, the observed number of genes in the overlap *O *is compared to its expected value


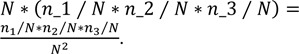


Transcription factor binding site (TFBS) Enrichment Analysis for H9- and Man12-derived HLCs treated with BPA, BPF and BPS was performed for up- and downregulated genesets in comparison to untreated controls, using oPOSSUM 3.0 single site analysis (SSA), taking into account JASPAR CORE profiles with a minimum specificity of 8 bits with a conservation cutoff of 0.4 in a region +/- 5kb around input genes. Motifs were ranked by z-score, representing the frequency of TFBS occurrence and Fisher-Test Scores were used for supporting interpretation (Ho Sui et al., 2005[18]). Gene ontology enrichment analysis was performed based on probe set IDs with the topGO package (Alexa and Rahnenführer, 2018[1]), using Fisher's exact test and the elim method. Only results from the biological process ontology were considered. The cutoff for the enrichment p value was set to 0.05. The microarray data is deposited in the GEO database, with accession number GSE160360 (https://www.ncbi.nlm.nih.gov/ geo/query/acc.cgi?acc=GSE160360).

### RT^2^ profile PCR array

Total RNA was isolated from hESC derived HLCs on day 18. This was reverse transcribed using the RT^2 ^First Strand Kit as per the manufacturer's instructions (Qiagen). Quantitative polymerase chain reactions (qPCR) were performed using the RT^2 ^Profiler PCR array system (Qiagen, catalog number 330231, GeneGlobal ID PAHS-021ZG) in a 384-well optical plate. Real time reactions were conducted on a Roche LightCycler 480 (Roche Life Sciences) and were performed in quadruplicate for each target gene. The gene expression was analyzed using the RT^2^ Profiler PCR array Data Analysis Spreadsheet (Qiagen, https://www.qiagen.com/us/resources/resourcedetail?id=b3396407-ecb5-4656-ac5d-5ea7b83a397e&lang=en).

### Statistical analysis

Unless indicated, all data were obtained from 5 replicates and are presented by mean ± standard deviation (SD).

## Results

Pluripotent stem cell hepatocyte differentiation was performed using two human embryonic stem cell (hESC) lines, H9 and Man12. Cells at the hepatoblast stage displayed cobblestone-like morphology and stained positive for the hepatoblast marker alpha-fetoprotein. At the end of the differentiation procedure, the hepatocyte-like cells (HLCs) exhibited hepatocyte morphology and expressed hepatocyte nuclear factor 4α (HNF4α) and albumin (ALB). In addition, HLCs possessed cytochrome P450 3A and 1A2 activity (Supplementary Figure 1). For the purposes of these experiments, we exposed hepatocyte progenitors to bisphenols to study their effects on the derivative HLCs. Hepatocyte specification and maturation was performed in the presence of BPA, BPF and BPS over a 6-point concentration range from of 0-500 μM. After 8 days of exposure, cell status was determined by measuring ATP levels and caspase activity. ATP depletion was most profound in BPA-treated cells with a half-maximal inhibitory concentration (IC_50_) of 250 μM for Man12 and 130 μM for H9 HLCs (Supplementary Figure 2). A similar response was observed with exposure to BPF although it appeared less toxic to both Man12 and H9 HLCs in comparison to BPA, with IC_50_ values of ~380 μM and ~265 μM, respectively (Supplementary Figure 2). Notably, BPS at the tested concentrations did not deplete ATP synthesis below 50 % in either cell line (Supplementary Figure 2). Measurement of caspase 3/7 activity was employed to study the balance between cell viability and programmed cell death (Supplementary Figure 3). Enzymatic activity was measured at the end of the hepatocyte specification process. At the ATP IC_50_ BPA did not induce any change in caspase activity in any of the hESC-derived HLCs and maximal caspase activity was observed at 250 μM BPA which was increased ~1.4 fold over controls in Man12 HLCs and ~2.1 fold in H9 HLCs. The exposure of Man12 and H9 HLCs to BPF (at the ATP IC_50_) did not induce an increase in caspase activity. Maximal activity was only observed at the highest tested BPF concentration in HLCs from both cell lines. Although exposure to BPS at the ATP IC_50_ did not induce an increase in H9 HLC caspase activity, Man12 HLC caspase activity increased with BPS concentration. 

**Figure 1:**
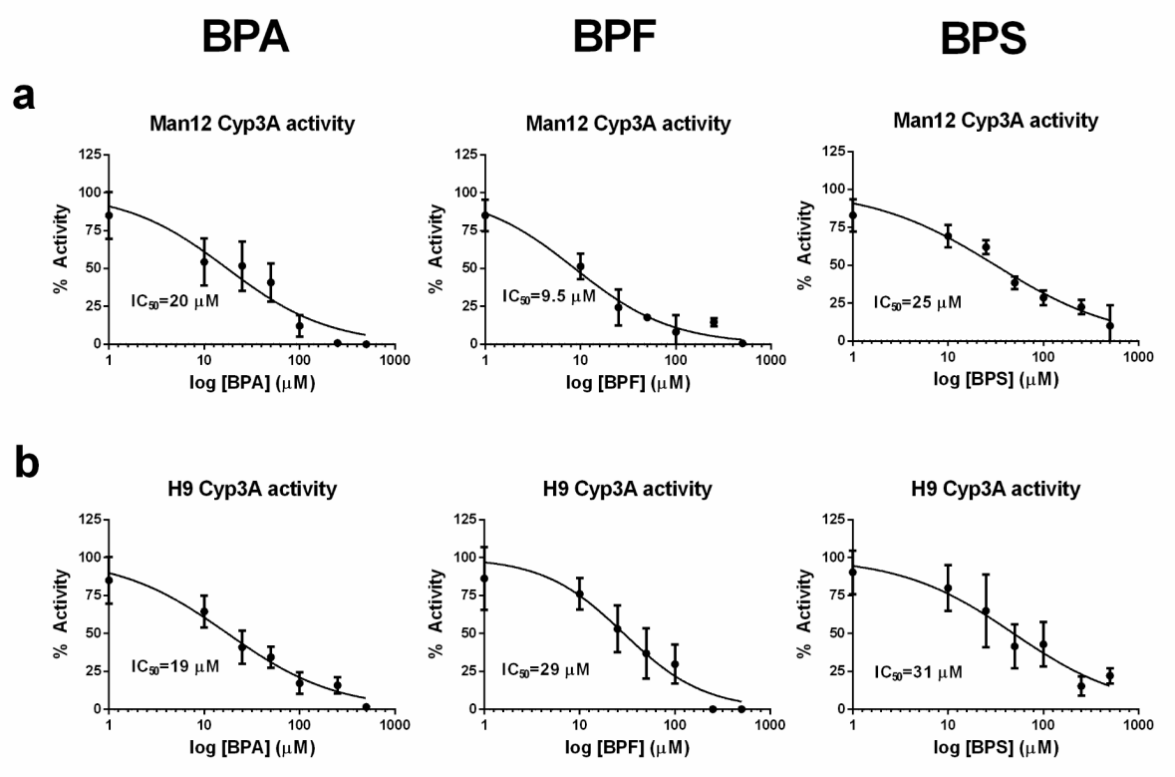
Concentration-response curves for cytochrome P450 3A (Cyp3A) activity in hepatocyte like cells following exposure to bisphenol A (BPA), bisphenol F (BPF) or bisphenol S (BPS), concentration range 0-500 µM. a. Cyp3A activity in Man12 HLCs. b. Cyp3A activity in H9 HLCs. Results are given as percentage and compared to the vehicle control. Mean values ± SD of *n=5 *replicates.

Following on from these experiments HLC function was assessed. We measured cytochrome P450 enzyme function which is an important marker of liver function. Analysis of Cyp3A P450 activity upon exposure to BPA revealed that both Man12 and H9 HLCs responded in similar fashion, with IC_50_ values of 20 μM and 19 μM respectively (Figure 1a(Fig. 1)). However, cell line differences were observed upon exposure to BPF and BPS. Man12 HLCs were slightly more sensitive to these compounds when compared to H9 HLCs. BPF- and BPS-exposed Man12 HLCs displayed IC_50_ values of ~10 μM and 25 μM respectively, whereas this was increased in H9 HLCs to ~29 μM and 31 μM respectively (Figure 1a, b(Fig. 1)). Cyp1A2 activity was also measured following exposure to the three bisphenols. In Man12 HLCs, BPA exposure resulted in an IC_50_ value of 51 μM. This was reduced in H9 HLCs to 20 μM (Supplementary Figure 3). When HLCs were exposed to BPF and BPS, Man12 Cyp1A2 IC_50_ values were 10 μM and 17 μM respectively. This was increased in H9 HLCs to 17 μM and 37 μM for BPF and BPS respectively (Supplementary Figure 4).

**Figure 2:**
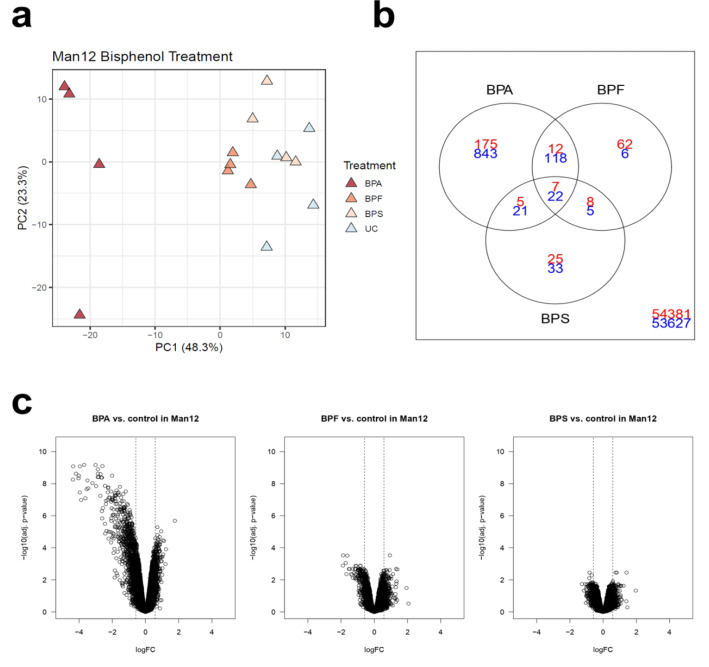
Principal component analysis of the 1000 genes with highest variance in Man12 HLCs treated with bisphenol A (BPA), bisphenol F (BPF) and bisphenol S (BPS) compared with untreated control (UC). a. The top principal components represent 70.6 % of the variance, *n=4* technical replicates. b. Venn diagram of DEGs in Man12 cells showing overlap between upregulated (red) and downregulated (blue) genes after treatment with different bisphenols. c. Volcano plots of DEGs for bisphenol treatments indicate BPA-induced deregulation in Man12 HLCs.

To obtain a global picture of changes in the HLC transcriptome following exposure to bisphenols, DNA microarray analysis was performed. Given the importance of Cyp3A function in fetal, neonatal and adult liver, we opted to use the Cyp3A IC_50_ value for each cell line and compared those to vehicle control populations. The lists of differentially expressed genes in Man12 and H9 derived HLCs are available in Supplementary Tables 1 and 2 and the corresponding GO enrichment analyses in Supplementary Tables 3 and 4 . Principal component analysis of Man12 HLCs showed a separation of BPA treated cells from the remaining populations on the first principal component, accounting for 48.3 % of the observed variance among the top 1000 differentially expressed genes (Figure 2a(Fig. 2)). Whereas Man12 HLCs exposed to BPF or BPS did not separate clearly from the untreated control (UC). This observation was also reflected in the number of uniquely deregulated probe set IDs. There is an increase in up- and downregulated probe set IDs for BPA treated cells (Figure 2b(Fig. 2)), as well as in the Volcano plot (Figure 2c(Fig. 2)), emphazising the highest observed fold changes among genes downregulated in response to BPA. 

**Figure 3:**
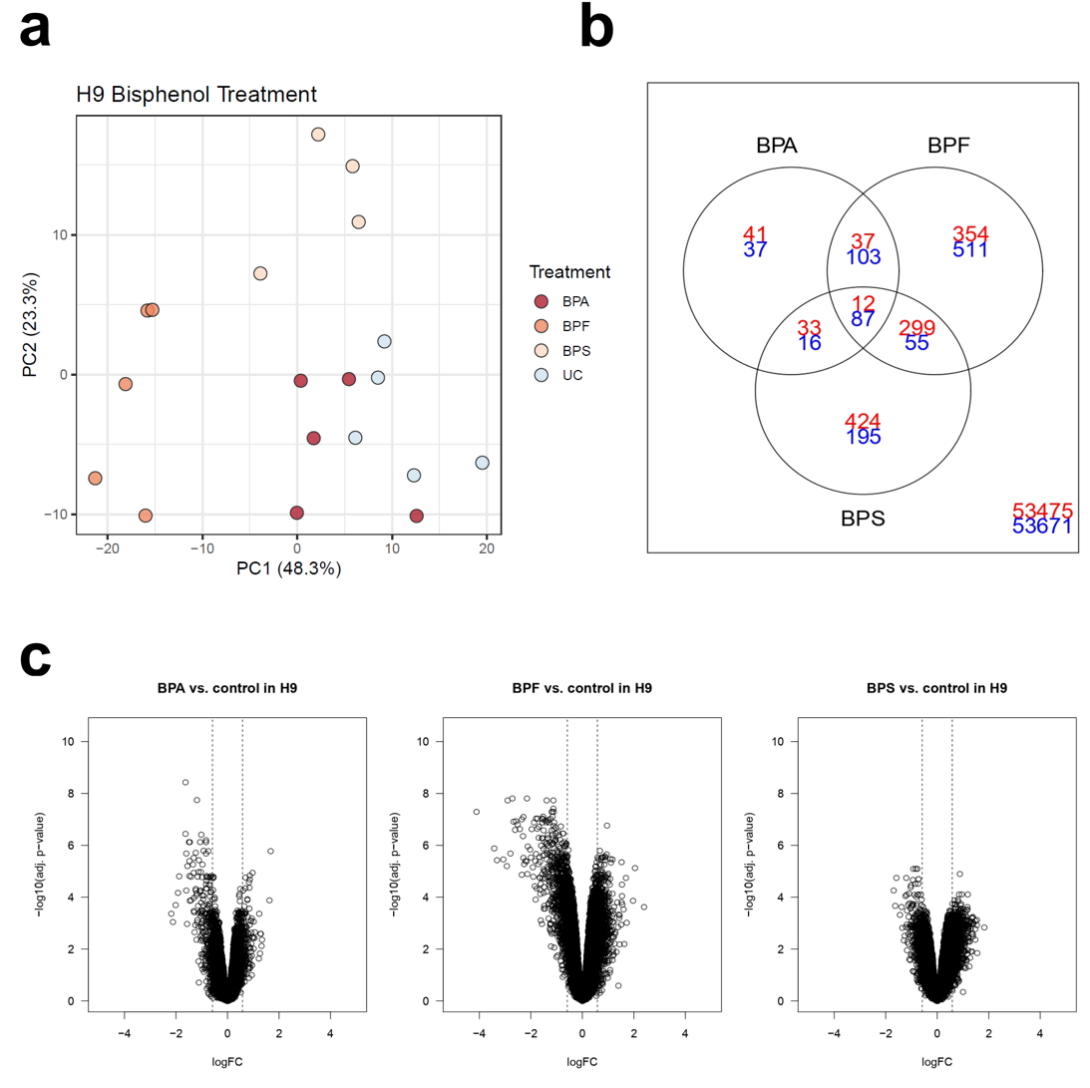
Principal component analysis of the 1000 genes with highest variance in H9 HLCs treated with bisphenol A (BPA), bisphenol F (BPF) and bisphenol S (BPS) compared to untreated control (UC). a. The top principal components represent 60.1 % of the variance, *n=4* replicates. b. Venn diagram of DEGs in H9 HLCs cells showing overlap between upregulated (red) and downregulated (blue) genes after treatment with different bisphenols. c. Volcano plots of DEGs for bisphenol treatments indicate BPF-induced deregulation in H9 HLCs.

In contrast, treatment of H9 HLCs with BPA did not lead to a clear separation of HLCs from the control population (Figure 3a(Fig. 3)). Instead, we could observe separation based on the first principal component of HLCs treated with BPF (40.5 % variance). In addition, BPS treatment led to separation on the second principal component accounting for 19.6 % of the variance, highlighting potential differences in response to these compounds, despite the extensive overlap among BPF and BPS deregulated probe sets (Figure 3b(Fig. 3)). Although we demonstrated changes in cell function, the H9 HLC transcriptome appeared less severely affected following BPA treatment. We also observed greater fold changes for downregulated genes in BPF treated H9 HLCs (Figure 3c(Fig. 3)).

**Figure 4:**
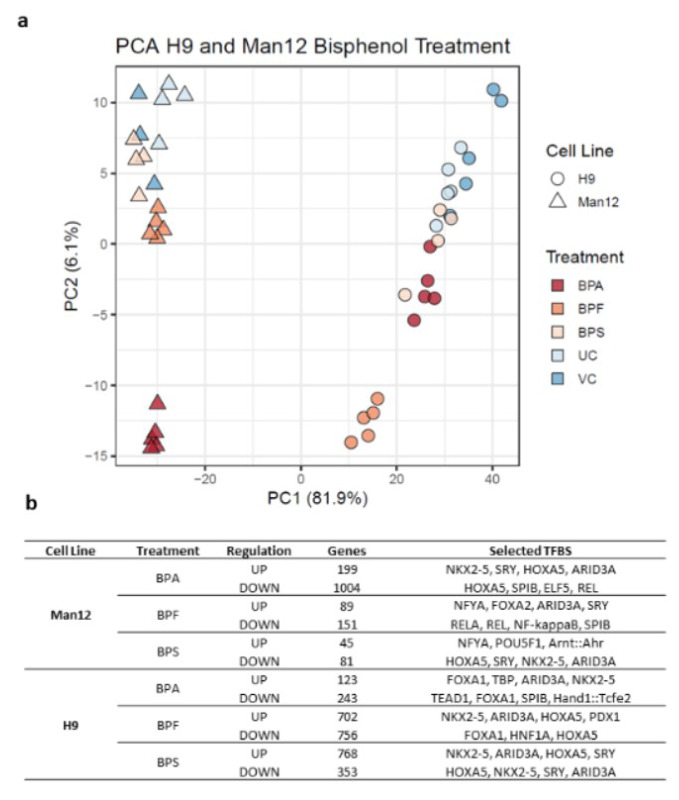
Principal component analysis of the 1000 genes and TFBS with highest variance in Man12 and H9 HLCs following treatment with bisphenols, compared to the vehicle control (VC) and untreated cells (UC) a. Principal component analysis of the 1000 genes with highest variance in H9 and Man12 HLCs treated with bisphenol A (BPA), bisphenol F (BPF) and bisphenol S (BPS) with vehicle control (VC) and untreated control (UC). The top principal components represent 88.0% of the variance, *n=4-5* replicates. b. Up- and downregulated genes and most relevant transcription factor binding sites (TFBS) in the three different cohorts are shown.

Principal component analysis of the 1000 genes with highest variance in H9 and Man12 HLCs treated with bisphenol A (BPA), bisphenol F (BPF) and bisphenol S (BPS) with VC and UC was performed (Figure 4a(Fig. 4)). We further examined overrepresented transcription factor binding sites using OPOSSUM, considering results with a p-value of smaller than 0.01 and Z-Score of at least the mean value plus the standard deviation of all Z-scores (a complete list of the results can be found in Supplementary Text Files 1-12).

Among genes upregulated in response to BPA exposure in Man12-derived HLC, overrepresented transcription factor binding sites (TFBSs) included NKX2.5, SRY and - although slightly less significant, but with high frequency of motif occurrence - HOXA5, ARID3a and Pdx1 (Figure 4b(Fig. 4), Supplementary Text File 1). These transcriptional regulators in part have been previously shown to be affected upon BPA exposure and play critical roles in regulating tissue-specific gene expression, differentiation and embryonic development (Huang et al., 2012[20]; Hussain, 2014[21]; Chang et al., 2016[5]; Ubuka et al., 2018[45]; Dou et al., 2019[9]). In line with the observed differences in gene expression variance (Figure 2a(Fig. 2)), induction of genes in H9 in response to BPA exposure was observed to a lower extent compared to Man12, leading to less significant enrichment of TFBSs among those genes, with FOXA1 enrichment dominating (Figure 4b(Fig. 4), Supplementary Text File 7). 

Among BPA downregulated genes, considerably overrepresented TFBS included ELF5, SPIB and REL which are known to play important roles in cell proliferation, survival and MAPK signaling (Figure 4b(Fig. 4), Supplementary Text File 2). While SPIB was also found overrepresented among downregulated genes in BPA exposed H9-derived HLCs, we found strong evidence for overrepresentation of TEAD1 which is implicated in cell cycle regulation, stemness and development (Meinhardt et al., 2020[31]) (Figure 4b(Fig. 4), Supplementary Text File 8). 

Following exposure to BPF, upregulated genes in Man12 HLCs were strongly enriched with TFBSs for NFYA, similar to ARID3A, implicated in the regulation of fat metabolism and reported to respond to BPA (Shu et al., 2019[38]) (Figure 4b(Fig. 4), Supplementary Text File 3). 

BPF upregulated genes in H9 also were enriched in ARID3A TFBSs, in addition to high frequency of binding sites for NKX2-5, HOXA5, PDX1 and SRY (Figure 4b(Fig. 4), Supplementary Text File 9). In line with BPA exposure, we observed downregulation of genes with TFBSs for REL and RELA in Man12 HLCs exposed to BPF and FOXA1 (Figure 4b(Fig. 4), Supplementary Text File 4). Likewise, in H9 HLCs most overrepresented TFBSs were associated FOXA1, but also HNF1A (Figure 4b(Fig. 4), Supplementary Text File 10). 

**Figure 5:**
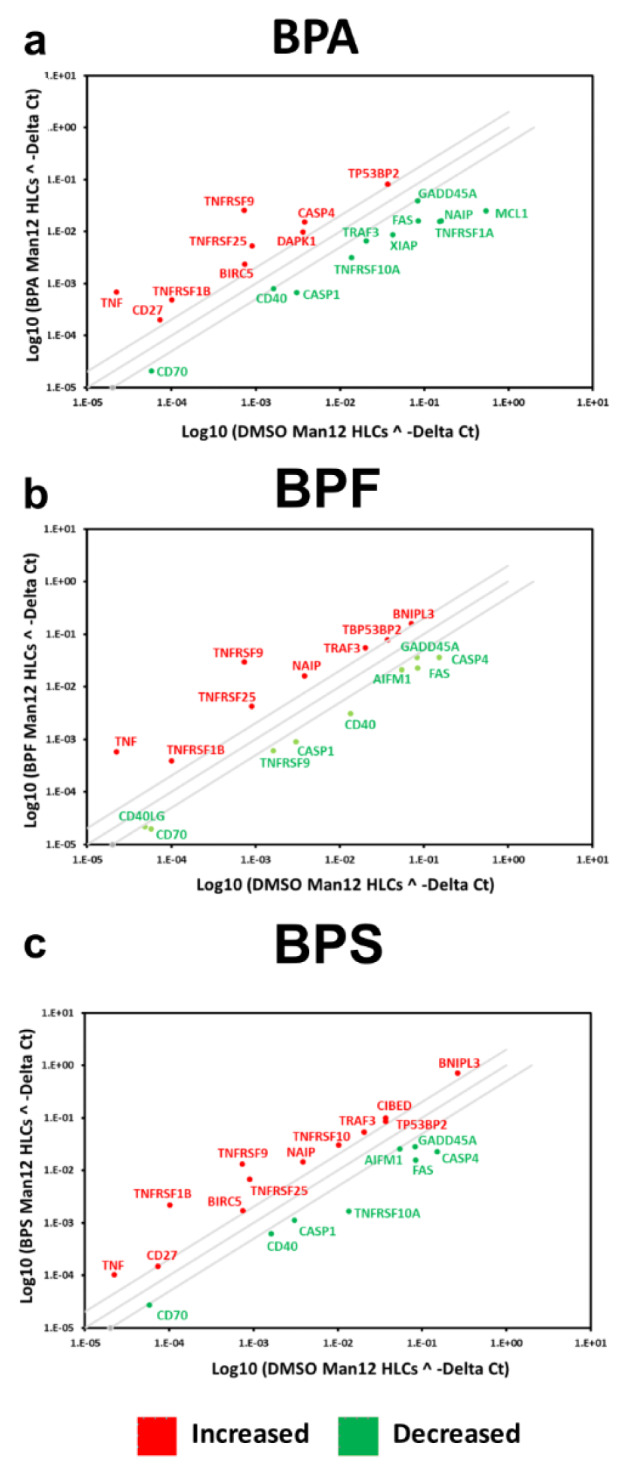
Differential gene expression of Man12 HLCs exposed to bisphenols. The scatter plots represent expression of the major genes expressed in human cells. The graph plots the log_10_ of normalized gene expression in control condition, DMSO treated Man12 HLCs versus experimental condition, Man12 HLCs (y axis) exposed to a bisphenol A (BPA), b bisphenol F (BPF) and c bisphenol S (BPS). Symbols outside the boundary area indicate fold differences larger than two folds. *n=4* replicates.

**Figure 6:**
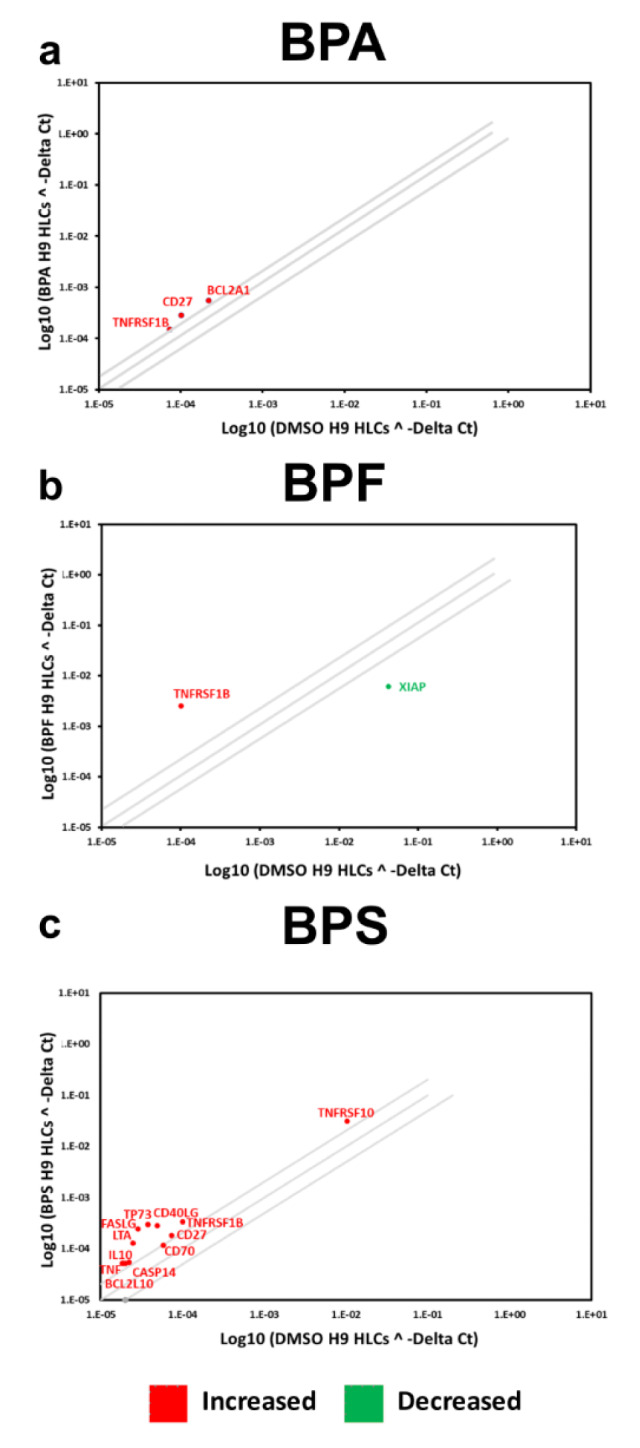
Differential gene expression of H9 HLCs exposed to bisphenols. The scatter plot of the major genes expressed in human cells. The graph plots the log_10_ of normalized gene expression in control condition, DMSO treated H9 HLCs versus experimental condition, H9 HLCs exposed to a bisphenol A (BPA), b bisphenol F (BPF) and c bisphenol S (BPS). Symbols outside the boundary area indicate fold differences larger than two folds. *n=4* replicates.

The PCR array experiments complemented those results with a shift in the balance of cell viability observed in HLCs exposed to BPA and BPF. In Man 12 HLCs exposed to BPA we observed increased expression of pro-apoptotic genes TNF, TNFRSF1B, TNFSF25, THNFRSF9, TP53BP2. This was paralleled with an increase in expression of anti-apoptotic genes BCL12A1, BIRC5, CD27, NAIP and DAPK1 (Figure 5a(Fig. 5), Supplementary Table 5). In Man 12 HLCs exposed to BPF we observed increased expression of pro-apoptotic genes TNF, TNFRSF25, TNFRSF9, TRAF3, TP53BP2, BCLI2A1 and anti-apoptotic genes BNIP3L, CD27 and NAIP (Figure 5b(Fig. 5), Supplementary Table 6). H9 HLCs exposed to BPA there was increased expression of pro and anti-apoptotic genes TNFRSF1B, CD27 and BCLI2A1 (Figure 6a(Fig. 6), Supplementary Table 8). Whereas exposure to BPF induced the expression of the pro-apoptotic death domain gene TNFRSF1B, and downregulated expression of the caspase inhibitor XIAP (Figure 6b(Fig. 6), Supplementary Table 9). Exposure to BPA and BPF manifested in significant levels of ATP depletion across the concentration curve, indicating that these changes in gene expression could have played an important role in reducing cell viability at the higher concentrations (Supplementary Figure 2).

Interestingly, BPS-exposed Man12 HLCs also demonstrated an upregulation of TFBSs for NFYA and Arnt::Ahr, which is involved in the regulation of xenobiotic metabolizing enzymes, xenobiotic induced toxicity and carcinogenesis (Barouki et al., 2007[3]) (Figure 4b(Fig. 4), Supplementary Text File 5). In H9 however, BPS exposure was very similar to BPF induced genes, with overrepresentation of NKX2-5, ARID3A, HOXA5 and SRY dominating the enrichment analysis (Figure 4b(Fig. 4), Supplementary Text File 11). In Man12 and H9, the same set of factors were observed to be enriched among downregulated genes, although with less confident enrichment in Man12 than H9 (Figure 4b(Fig. 4), Supplementary Text Files 6 and 12). 

In Man 12 HLCs exposed to BPS, the PCR array experiments also provided evidence that there was a shift in the balance of cell viability, with increased expression of pro-apoptotic genes TNF, CIDEB, TNFRSF25, TNFRSF9, TNFSF10, TP53BP2, and TRAF3. Whereas anti-apoptotic genes CD27, BIRC5, BNIP3 and NAIP were upregulated (Figure 5c(Fig. 5), Supplementary Table 7). In H9 HLCs increased expression of the pro-apoptotic genes TNF, CASP14, CD70, FASLG, LTA and TNFRSF10 was observed paralleled with an increase in anti-apoptotic gene expression, BCL2L10, CD27, CD40LG and TP73 (Figure 6c(Fig. 6), Supplementary Table 10). Exposure to BPS did not manifest in significant ATP depletion across the concentration curve, indicating that these changes in gene expression did not lead to dramatic changes in cell viability (Supplementary Figure 2).

**Figure 7:**
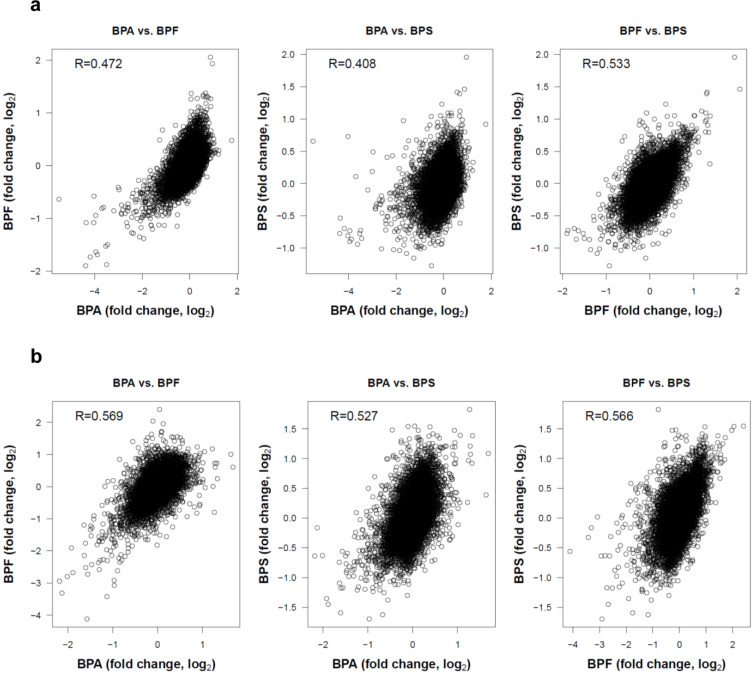
Pairwise correlations of gene expression changes (expressed as log_2_ fold changes) of the three bisphenol derivatives BPA, BPF and BPS. The Spearman correlation coefficients (R) are given. P-values of all pairwise comparisons were smaller than2.2 × 10^-16^. a. Man12 derived HLCs; b. H9 derived HLCs.

Finally, we studied the degree of similarity of the gene expression changes induced by BPA, BPF and BPS. For this purpose, pairwise correlations between BPA and the two other derivatives were studied (Figure 7(Fig. 7)). In Man12 derived HLCs the Spearman correlation coefficients (R) ranged between 0.408 and 0.533 (Figure 7a(Fig. 7)). The corresponding correlation coefficients in H9 derived HLCs were between 0.527 and 0.569 (Figure 7b(Fig. 7)). The p-values of all pairwise comparisons were very small (<2.2 × 10^-16^). These analyses demonstrate a high degree of correlation of the gene expression changes induced by the three bisphenol derivatives.

Moreover, we analyzed the degree of overlap of the genes up- or downregulated in pairwise comparisons of BPA, BPF and BPS (Table 1(Tab. 1)). If two bisphenol derivatives share a certain number of deregulated genes, it can be calculated by which factor the overlapping deregulated genes exceed the randomly expected number. An overlap ratio of one means that the number of genes in the overlap is exactly as randomly expected based on the number of differential genes and the total number of analyzed genes. The results demonstrate a very high degree of overlap of the genes whose expression was influenced by the three compounds. Even the lowest overlap ratio of all pairwise comparisons demonstrated that 28.9-fold more genes, than randomly expected, were downregulated by both BPA and BPS.

## Discussion

In this report we used hepatocyte-like cells, derived from pluripotent stem cells, to study the effects of exposure to BPA, BPF and BPS. Stem cell-derived HLCs can be produced from defined genetic backgrounds and represent an unlimited source of somatic cells for use in modeling studies (Rashid et al., 2010[33]; Si-Tayeb et al., 2010[39]; Hannan et al., 2013[14]; Szkolnicka et al., 2014[42], 2016[43]; Rashidi et al., 2016[34], 2018[35]; Touboul et al., 2016[44]; Lucendo-Villarin et al., 2017[27], 2019[29], 2020[28]; Wang et al., 2019[50]). Although HLCs exhibit major differences compared to primary human hepatocytes, they can be used to gain an overview of the toxic effects of structurally related compounds. 

Exposure of these cells to bisphenols induced expression changes in genes associated with cellular differentiation, embryonic development, proliferation, survival and apoptosis (summarized in Table 2(Tab. 2) and Table 3(Tab. 3)). Genes dysregulated by BPA, BPF and BPS showed a large overlap. The overlap ratio for three compounds was calculated as the observed number of genes in the overlap divided by the expected number of genes in this overlap under the assumption of independence. Independence means that for each compound a set of genes is randomly drawn from all genes, where the set is of the same size as the set of differential genes for the respective compound. The overlap ratio for genes up- or downregulated by all three compounds in H9 derived HLC was 540.9 (12 upregulated genes compared to 0.02218 expected) and 4010.5 (87 downregulated genes compared to 0.02169 expected). For Man12 derived HLCs the corresponding numbers were 26255.4 (7 upregulated genes compared to 0.00027 expected) and 5355.6 (12 downregulated genes compared to 0.00410 expected). These results indicate that BPA, BPF and BPS induced relatively similar gene expression alterations in the investigated cell types (Figure 7(Fig. 7), Table 1(Tab. 1)). 

For gene array analysis, we used the IC_50_ values of the inhibition of cytochrome P450 3A activity, which were 19, 29 and 31 µM for BPA, BPF and BPS, respectively, in H9 derived HLCs. The corresponding IC_50_ values in Man12 derived HLCs were 20 µM (BPA), 9.5 µM (BPF) and 25 µM (BPS), suggesting no major differences of the three bisphenol derivatives. It should be considered that the concentrations used in this study are much higher compared to BPA serum concentrations of the general population. Biomonitoring studies using LC/MS-MS and controlling for possible contaminations usually reported free, non-conjugated bisphenol A concentrations smaller than 1 µg/L in blood or serum (corresponding to < 4.4 nM (review: Hengstler et al., 2011[16]). Here, we used higher concentrations, because we aimed for a comparison of gene expression alterations induced by the three bisphenol derivatives. Since estrogen sensitive tissues (and other possible target tissues) are exposed to more than 1000-fold lower BPA concentrations, it can be expected that *in vivo* no gene expression changes will be induced by BPA nor its derivatives.

In the global transcriptomic studies, Man12 HLCs showed a larger transcriptional response to BPA (Figure 2(Fig. 2)), whereas H9 HLC exposure to BPF resulted in the greatest transcriptional response (Figure 3(Fig. 3)). Following on from these studies, we analyzed transcription factor binding sites (TFBS) that were either up- or downregulated following exposure to the BPs (Figure 4(Fig. 4)). We observed that exposure to BPA in Man12 HLCs induced an increase in the representation of TFs associated with tumor suppression via p53. This included ARID3A, NKX3-1 and HOX5A (Raman et al., 2000[32]; Lei et al., 2006[25]; Lestari et al., 2012[26]) and the inhibition in the expression of REL, a subunit of the NF-kB complex involved in cell signaling and survival (Hay et al., 2001[15]; Gilmore, 2006[11]). We also observed decreased representation of the epithelial determination factor, ELF5. Upon exposure of Man12 HLCs to BPF we observed an increase in cell identity transcription factors including TBP, FOXA1, FOXA2 but also MEF2, a protein that promotes epithelial to mesenchymal transition during the onset of hepatocellular carcinoma (Yu et al., 2014[55]). As before, we also observed a decrease in NF-kB family member representation, REL and RELA (Gilmore, 2006[11]). BPF exposure also resulted in a decrease in the representation of SPIB, a transcription factor regulator of tumor progression whose expression can be used to stage liver cancer (Ho et al., 2016[19]). BPS exposure of Man12 HLCs induced an upregulation in the representation of ZNF143, a transcription factor that regulates C/EBPa expression important in the fetal liver (Ye et al., 2013[54]; Gonzalez et al., 2017[13]). BPS also induced the expression of ARID3 and IRF1. Whereas a loss of tissue specification and cell identity transcription factors; HOX5A, FOXA1, FOXD3 and FOXL1 were detected. 

Following H9 HLC exposure to the three bisphenols we observed an upregulation in the representation of NKX5.2, a conserved homeobox required for protection against the stress-induced apoptosis in fetal liver (Kasahara et al., 1998[23]) and cardiomyocytes (Zheng et al., 2013[58]). This was corroborated by the increase in the p53 mediator ARID3. We also observed an increased representation of transcription factors associated with tissue specific and cell identify including; FOXA1 and FOXA2 in response to BPA and FOXD3 (Zaret et al., 2008[56]) in response to BPF and BPS. Interestingly, BPA exposure led to a decrease in the representation of the heterodimer HAND1::TCFE2A, whose depletion has been reported to promote cell apoptosis (Andrysik et al., 2013[2]). We also observed reduced KLF4 representation, a transcription factor that controls the cell cycle progression following DNA damage and activation by p53 (Zhang et al., 2000[57]), indicating it may suppress apoptosis (Yamanaka, 2007[52]). Moreover, KLF4 possesses a crucial role in somatic cell reprogramming and pluripotent stem cell self-renewal (Kim et al., 2008[24]). In response to BPF and BPS, H9 HLCs displayed reduced representation of the liver cancer marker SPIB. In response to BPS, we observed the decreased representation of the CCCTC-binding factor (CTCF), a key regulator of hepatocyte proliferation in response to injury (Wang et al., 2020[48]) and NFATC2, which is enriched in chromatin-accessible regions of genes involved in the development of liver steatosis and hepatocellular carcinoma (Dechassa et al., 2018[7]). 

A consistent readout from the DNA microarray analysis were transcription factors involved in cell viability and apoptosis. Therefore, we employed a focussed PCR array to examine key genes involved in cell viability and apoptosis (Figure 5(Fig. 5) and 6(Fig. 6) and Supplementary Tables 5-10 ). Exposure of Man12 HLCs to all bisphenols resulted in the activation of apoptotic and inflammatory gene expression associated with the TNF signaling pathway; TNF receptors TNFRSF1B, TNFSF25 and TNFRSF9 supporting a pro-apoptotic environment (Seachrist et al., 2016[37]). Of note, we also observed increased expression of anti-apoptotic genes, demonstrating significant changes in signaling equilibria took place following bisphenol exposure (Figure 5(Fig. 5)). The upregulation of the apoptosis regulator NAIP, also known as BIRC1, suggested a novel pathway induced by bisphenols in hepatocytes. In addition, the increased expression of two further anti-apoptotic genes, BIRC5 and CD27, were detected in Man12 HLCs following exposure to BPA and BPS (Figure 5a, c(Fig. 5)). 

Independent of the HLC tested, BPA promoted upregulation of the anti-apoptotic gene BCL2A1 which, prevents the release of pro-apoptotic cytochrome C from mitochondria and reduces caspase activation (Kale et al., 2018[22]). This suggests a common cellular mechanism to compensate the apoptotic signals triggered by BPA (Figure 5a(Fig. 5), 6a(Fig. 6)). In support of this, BCL2L10 expression, was also increased in H9 HLCs exposed to BPS (Figure 6c(Fig. 6)). As previously observed in Man12 HLCs, exposure of H9 HLCs to BPA or BPS induced CD27 expression (Figure 6a, c(Fig. 6)), suggesting that these two bisphenols activate a similar anti-apoptotic response. H9 HLCs exposed to BPS also displayed increased expression of the anti-apoptotic genes CD40LG and TP73 (Yao et al., 2019[53]) (Figure 6c(Fig. 6)). As with previous BPs, BPS also induced the expression of pro-apoptotic genes. In H9 HLCs the TNF signaling pathway was activated and we detected increased expression in TNF receptors TNFRSF1B and TNFSF10; LTA; Caspase 14; CD27 ligand or CD70 and the TNF activated FasLG pathway, (Faletti et al., 2018[10]) (Figure 6c(Fig. 6)). Expression of the TNF receptor TNFRS1B was commonly upregulated in response to all bisphenols tested and in both cell lines. It has recently been reported that low doses of BPA induced upregulation of this receptor in human placental cells (de Aguiar Greca et al., 2020[6]). 

In conclusion, we demonstrate that bisphenol exposure perturbates cytochrome P450 function and gene expression with relatively similar effects. Exposure of HLCs to bisphenols induced changes in the pathways associated with cellular differentiation, embryonic development, cell proliferation, survival and apoptosis. Taken together, our stem cell-based model provides a comprehensive overview of the effects of human bisphenol exposure in the developing liver and provides credible leads for future *in vitro* experimentation.

## Acknowledgements

BLV and DCH were funded by an award from the Chief Scientist Office (TCS 16/37). This work has received funding from the European Union’s Horizon 2020 research and innovation programme under grant agreement no. 681002 (EU-ToxRisk) and from TransQST (no. 116030).

## Disclosure

Professor David C Hay is a founder, shareholder and director of Stemnovate Limited.

## Supplementary Material

supplementary_tables_11_Antibodies

Supplementary material

supplementary_tables_1A_Differential genes, Man12 upregulated

supplementary_tables_1B_Differential genes, Man12 downregulated

supplementary_tables_2A_Differential genes, H9 upregulated

supplementary_tables_2B_Differential genes, H9 downregulated

supplementary_tables_3A_GO analysis, Man12 upregulated

supplementary_tables_3B_GO analysis, Man12 downregulated

supplementary_tables_4A_GO_analysis_H9 up

supplementary_tables_4B_GO_analysis_H9 down

supplementary_text_file_1_Signature upregulation BPA Man 12

supplementary_text_file_7_Signature upregulation BPA H9

supplementary_text_file_2_ Signature downregulation BPA Man 12

supplementary_text_file_8_Signature downregulation BPA H9

supplementary_text_file_3_Signature upregulation BPF Man 12

supplementary_text_file_9 Signature upregulation BPF H9

supplementary_text_file_4_Signature downregulation BPF Man 12

supplementary_text_file_10_Signature downregulation BPF H9

supplementary_tables_5_M12_BPA

supplementary_tables_6_H9_BPA

supplementary_tables_8_H9_BPF

supplementary_tables_9_M12_BPS_repariert

supplementary_text_file_5_Signature upregulation BPS Man 12

supplementary_text_file_11_Signature upregulation BPS H9

supplementary_text_file_6_Signature downregulation BPS Man 12

supplementary_text_file_12_Signature downregulation BPS H9

supplementary_tables_7_M12_BPF

supplementary_tables_10_H9_BPS

## Figures and Tables

**Table 1:**
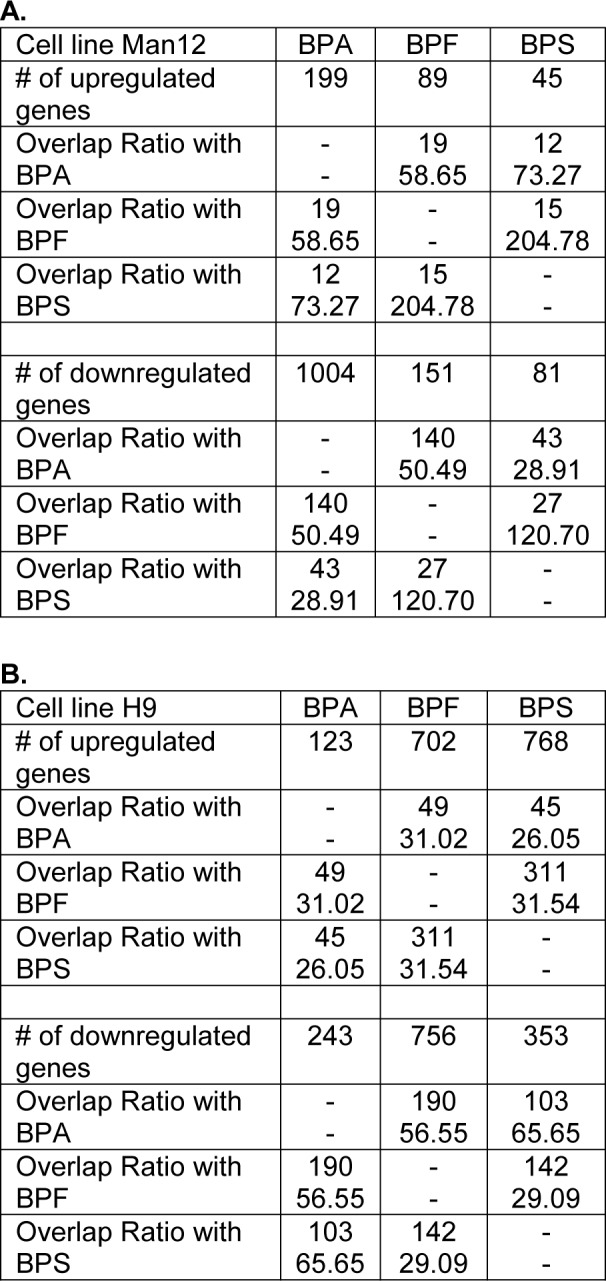
Overlap ratios of differentially expressed genes for pairwise comparison of BPA, BPF and BPS in Man12 (A) derived and H9 (B) derived HLCs. For each pairwise comparison the number of up- or downregulated genes (upper field) and the overlap ratio (lower field) is given. Moreover, the total number of deregulated genes (e.g. # of upregulated genes) is listed.

**Table 2:**
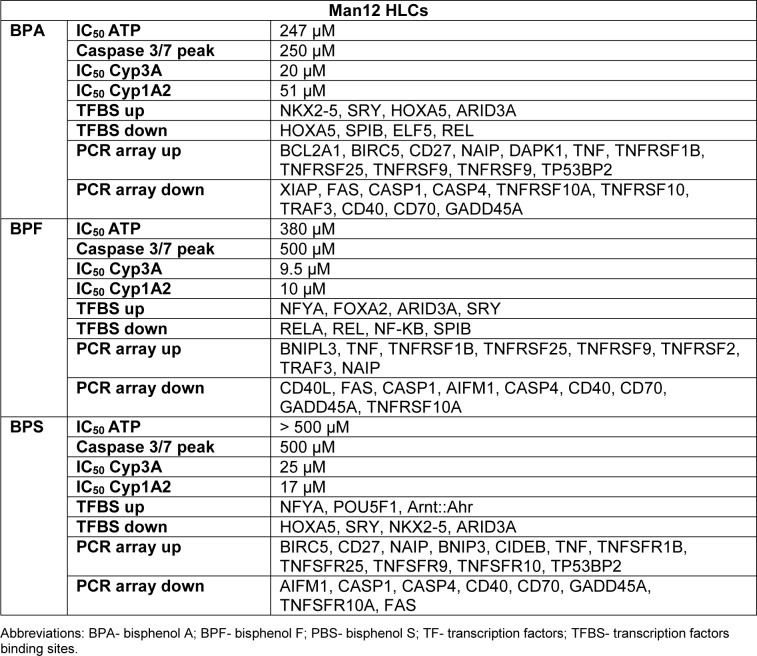
Summary of the Man12 HLCs exposure to bisphenols

**Table 3:**
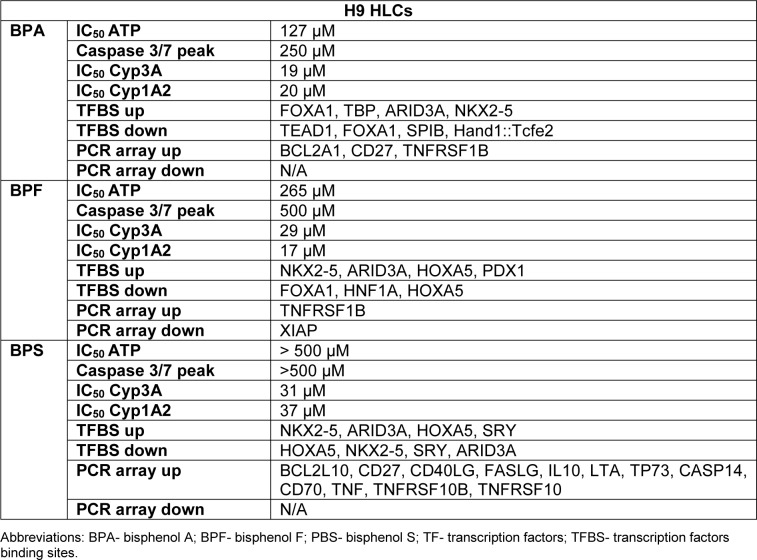
Summary of the H9 HLCs exposure to bisphenols
