# Supplementary material for: Genome-wide expression changes induced by bisphenol A, F and S in human stem cell derived hepatocyte-like cells
Source: EXCLI J. 2020 Nov 4;19:1459–76. doi: 10.17179/excli2020-2934 (PMC7726493; doi:10.17179/excli2020-2934)
Supplement: supplementary_tables_11_Antibodies [file EXCLI-19-1459-s-001.docx]

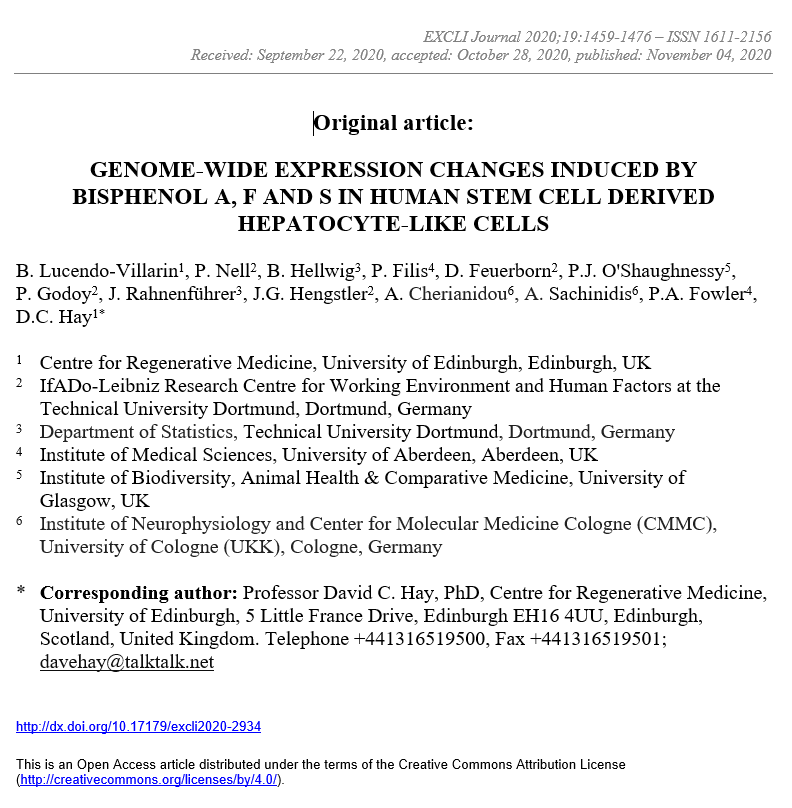


**Supplementary Table 11:** Antibodies employed in immunofluorescence studies

| **Antigen** | **Manufacturer** | **Host** | **Dilution** |
| --- | --- | --- | --- |
| HNF4α | Santa Cruz | Rabbit | 1:100 |
| Albumin | Abcam | Mouse | 1:100 |
| Alpha-fetoprotein | Abcam | Mouse | 1:500 |
| IgG | DAKO | Rabbit | 1:400 |
| IgG | DAKO | Mouse | 1:400 |
| Alexa Fluor 568 anti rabbit | Life Technologies | Donkey | 1:400 |
| Alexa Fluor 488 anti mouse | Life Technologies | Goat | 1:400 |
