# Supplementary material for: Genome-wide expression changes induced by bisphenol A, F and S in human stem cell derived hepatocyte-like cells
Source: EXCLI J. 2020 Nov 4;19:1459–76. doi: 10.17179/excli2020-2934 (PMC7726493; doi:10.17179/excli2020-2934)
Supplement: Supplementary material [file EXCLI-19-1459-s-002.pdf]

EXCLI Journal 2020;19:1459-1476 – ISSN 1611-2156
Received: September 22, 2020, accepted: October 28, 2020, published: November 04, 2020

Supplementary material to:

GENOME-WIDE EXPRESSION CHANGES INDUCED BY BISPHENOL A, F AND S IN HUMAN STEM CELL DERIVED HEPATOCYTE-LIKE CELLS

B. Lucendo-Villarin[1], P. Nell[2], B. Hellwig[3], P. Filis[4], D. Feuerborn[2], P.J. O'Shaughnessy[5], P. Godoy[2], J. Rahnenführer[3], J.G. Hengstler[2], A. Cherianidou[6], A. Sachinidis[6], P.A. Fowler[4], D.C. Hay[1*]

[1] Centre for Regenerative Medicine, University of Edinburgh, Edinburgh, UK
[2] IfADo-Leibniz Research Centre for Working Environment and Human Factors at the Technical University Dortmund, Dortmund, Germany
[3] Department of Statistics, TU Dortmund University, Dortmund, Germany
[4] Institute of Medical Sciences, University of Aberdeen, UK
[5] Institute of Biodiversity, Animal Health & Comparative Medicine, University of Glasgow, G61 1QH, UK
[6] Institute of Neurophysiology and Center for Molecular Medicine Cologne (CMMC), University of Cologne (UKK), Cologne, Germany

* **Corresponding author:** Professor David C. Hay, PhD, Centre for Regenerative Medicine, University of Edinburgh, 5 Little France Drive, Edinburgh EH16 4UU, Edinburgh, Scotland, United Kingdom. Telephone +441316519500, Fax +441316519501; davehay@talktalk.net

http://dx.doi.org/10.17179/excli2020-2934

This is an Open Access article distributed under the terms of the Creative Commons Attribution License (http://creativecommons.org/licenses/by/4.0/).

EXCLI Journal 2020;19:1459-1476 – ISSN 1611-2156
Received: September 22, 2020, accepted: October 28, 2020, published: November 04, 2020

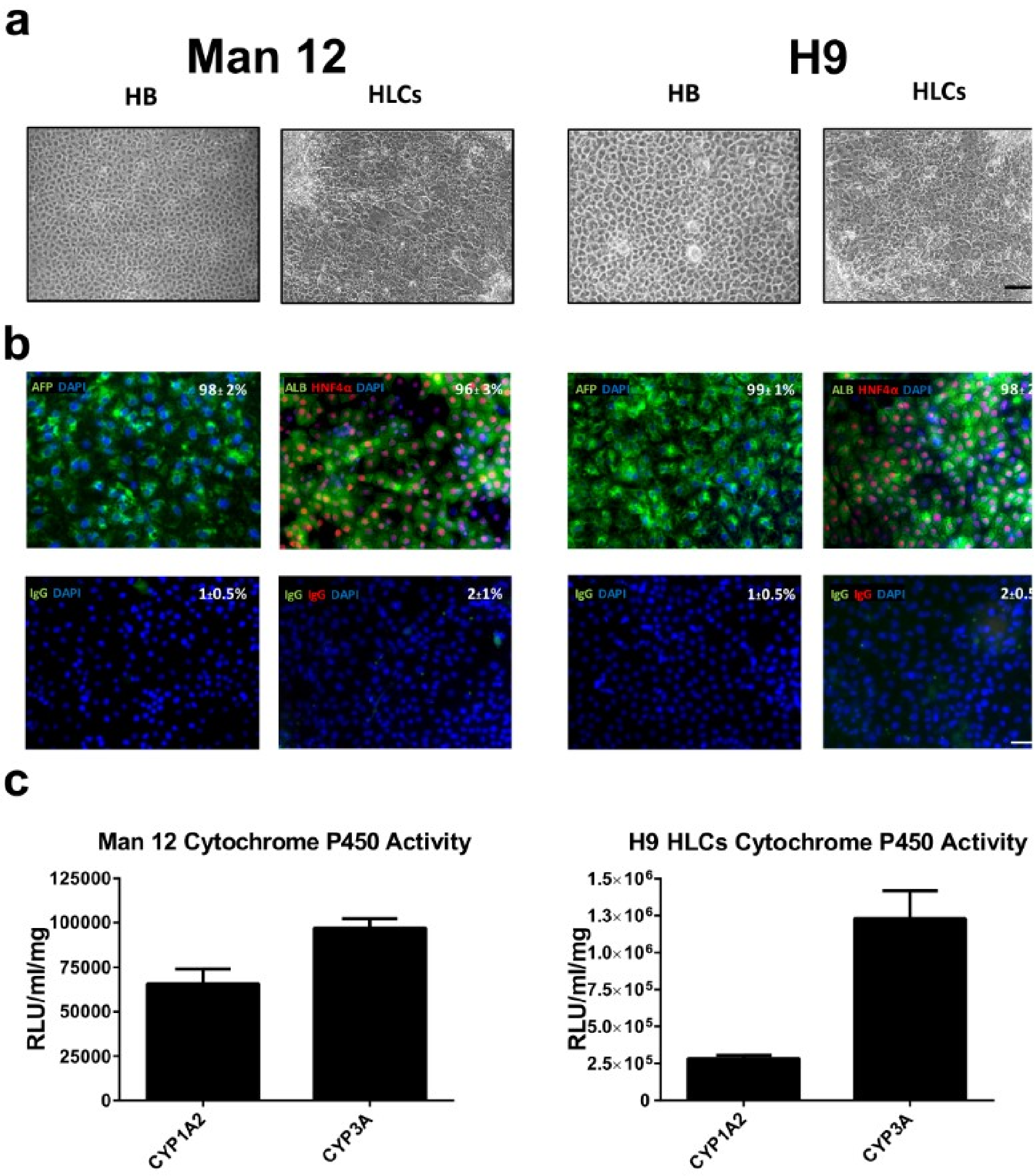

Supplementary Figure 1: Characterization of human stem cell derived hepatocytes. **a** During hepatocyte differentiation hESC Man12 and H9 displayed characteristic hepatoblast and hepatocyte morphology. Morphological images were taken at x10 magnification and scale bars represent 200 µm. **b** Immunofluorescence was employed to examine the expression of liver markers alpha-fetoprotein (day 10) and HNF4α (red) and albumin (green) on day 18. Negative controls were performed with corresponding immunoglobulin G. The percentage of positive cells is provided in the top right of each channel. This was calculated from 8 fields of view and is shown as mean values ± SD. Images were taken at x20 magnification and scale bars represent 100 µm. **c** Stem cell derived hepatocytes displayed Cytochrome P450 function. Results represent the mean values ± standard deviation (SD). *n=3*. Abbreviations: HB, hepatoblast; HLCs, hepatocyte like-cells; AFP, alpha-fetoprotein; ALB, albumin; HNF4α, hepatocyte nuclear factor 4α, DAPI, 4',6'-diaminido-2-phenylindole; IgG, immunoglobulin G; RLU, relative light units.

EXCLI Journal 2020;19:1459-1476 – ISSN 1611-2156
Received: September 22, 2020, accepted: October 28, 2020, published: November 04, 2020

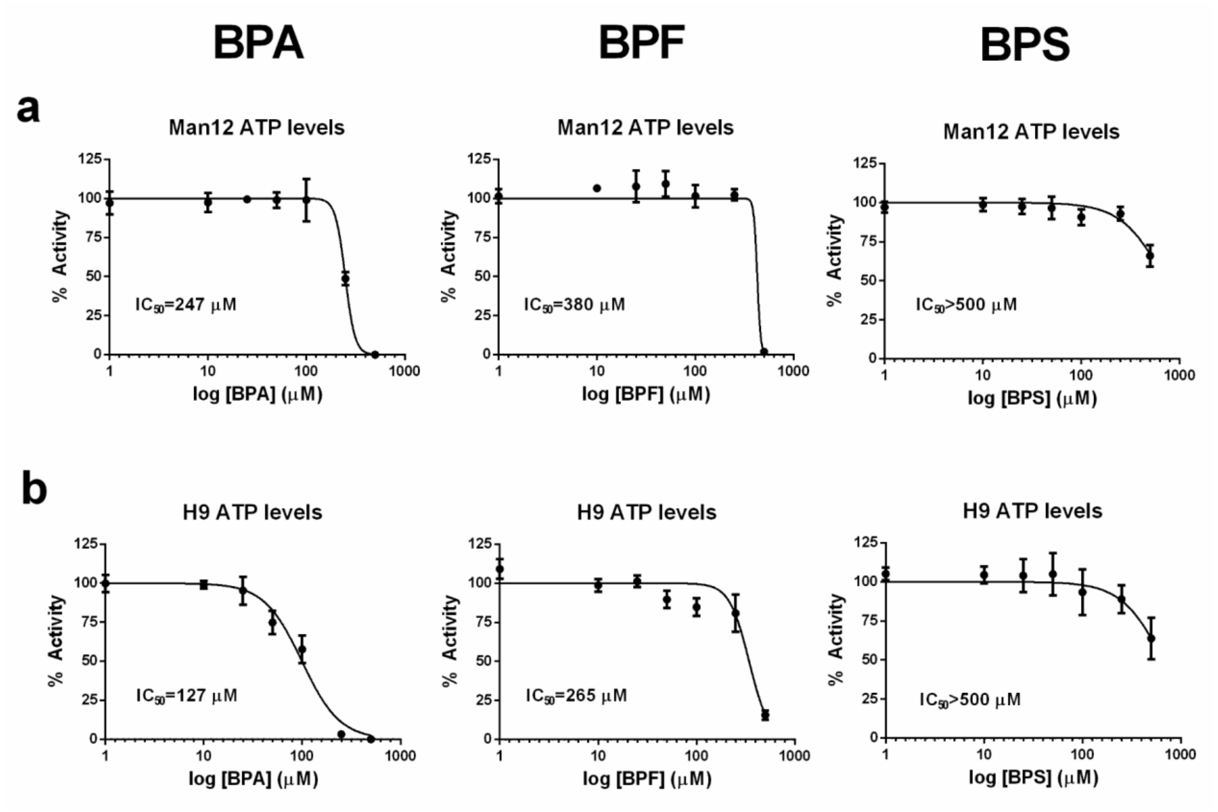

Supplementary Figure 2: Concentration-response curves for ATP levels in hepatocyte-like cells (HLCs) following exposure to a concentration range (0-500uM) of bisphenol A (BPA), bisphenol F (BPF) or bisphenol S (BPS). **a** ATP measurement in Man12 HLCs. **b** ATP measurement in H9 HLCs. The IC_{50} for each compound is provided. Results are given as percentage compared to the vehicle control. Mean values ± SD of *n=5* replicates.

EXCLI Journal 2020;19:1459-1476 – ISSN 1611-2156
Received: September 22, 2020, accepted: October 28, 2020, published: November 04, 2020

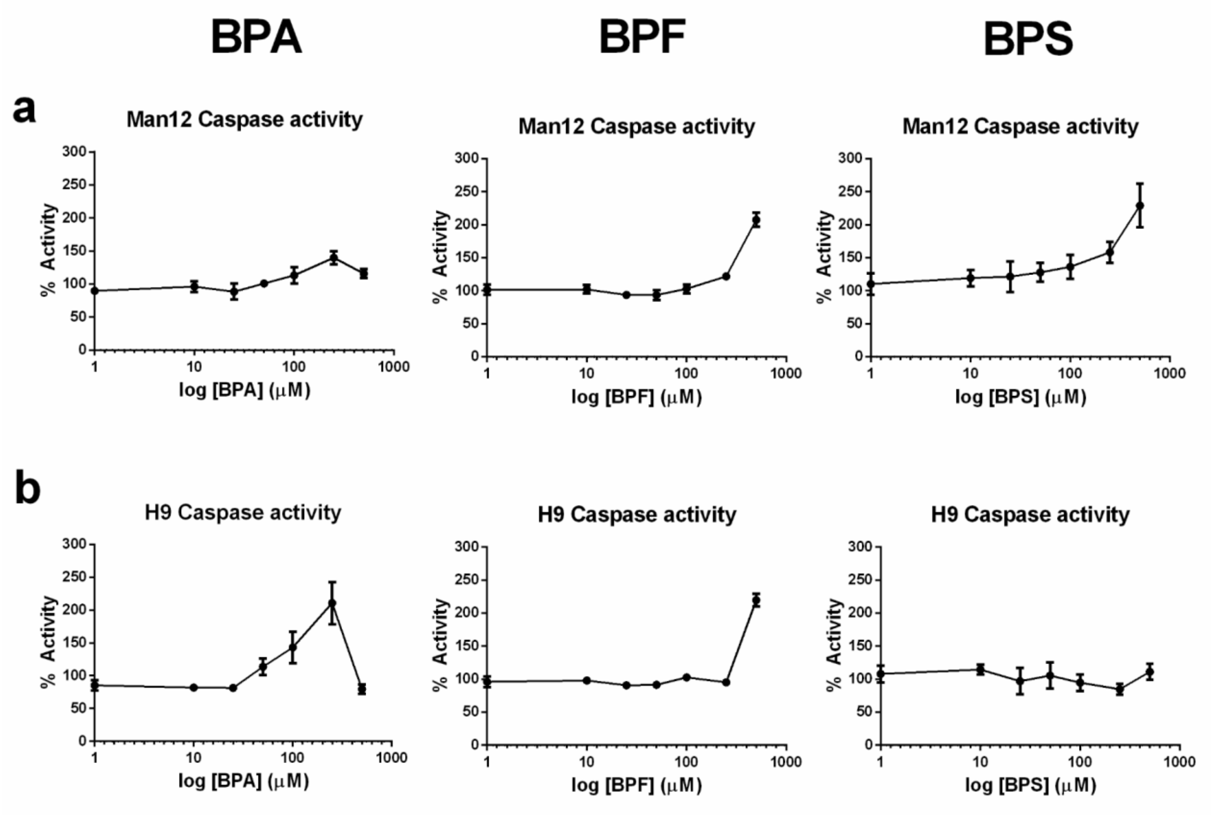

Supplementary Figure 3: Concentration-response curves for caspase 3/7 activity in hepatocyte like cells (HLCs) after 8 days of exposure to a concentration range (0-500 µM) of bisphenol A (BPA), bisphenol F (BPF) or bisphenol S (BPS). **a.** Caspase 3/7 measurement in Man12 HLCs. **b.** Caspase 3/7 measurement in H9 HLCs. Results are the mean of five replicates ± SD. Results are given as percentage compared to the vehicle control. Mean values ± SD of *n=5* replicates.

EXCLI Journal 2020;19:1459-1476 – ISSN 1611-2156
Received: September 22, 2020, accepted: October 28, 2020, published: November 04, 2020

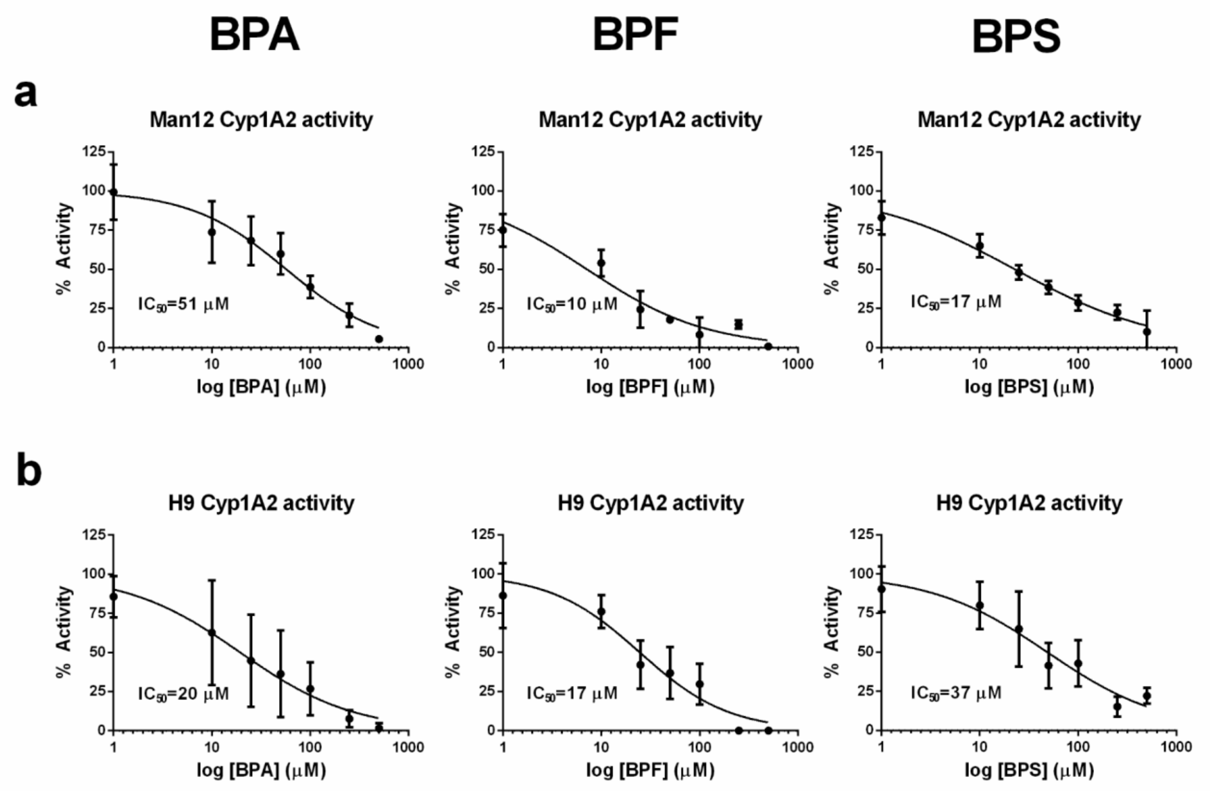

Supplementary Figure 4: Concentration-response curves for cytochrome P450 1A2 (Cyp1A2) activity in hepatocyte like cells (HLCs) following exposure to bisphenol A (BPA), bisphenol F (BPF) or bisphenol S (BPS). **a.** Cyp1A2 activity in Man12 derived HLCs. **b.** Cyp1A2 activity in H9 HLCs. Results are given as percentage compared to the vehicle control. Mean values ± SD of *n=5* replicates.

Supplementary Text File 1. Signature upregulation BPA Man 12

Supplementary Text File 2. Signature downregulation BPA Man 12

Supplementary Text File 3. Signature upregulation BPF Man 12

Supplementary Text File 4. Signature downregulation BPF Man 12

Supplementary Text File 5. Signature upregulation BPS Man 12

Supplementary Text File 6. Signature downregulation BPS Man 12

Supplementary Text File 7. Signature upregulation BPA H9

Supplementary Text File 8. Signature downregulation BPA H9

Supplementary Text File 9. Signature upregulation BPF H9

Supplementary Text File 10. Signature downregulation BPF H9

Supplementary Text File 11. Signature upregulation BPS H9

Supplementary Text File 12. Signature downregulation BPS H9

Supplementary Table 1A. Differential genes, Man12 upregulated

Supplementary Table 1B. Differential genes, Man12 downregulated

Supplementary Table 2A. Differential genes, H9 upregulated

Supplementary Table 2B. Differential genes, H9 downregulated

Supplementary Table 3A. GO analysis, Man12 upregulated

Supplementary Table 3B. GO analysis, Man12 downregulated

Supplementary Table 4A. GO analysis, H9 upregulated

Supplementary Table 4B. GO analysis, H9 downregulated

Supplementary Table 5. Fold change (2^(- Delta Delta Ct)) of the normalized gene expression (2^(- Delta Ct)) in the Test Sample (BPA treated Man12 HLCs) divided by the normalized gene expression (2^(- Delta Ct)) in the Control Sample (DMSO treated Man12 HLCs). Fold regulation represents fold change results in a biologically meaningful way. Fold change values greater than one indicate a positive- or an upregulation, and the fold regulation is equal to the fold change. Fold change values less than one indicate a negative or downregulation, and the fold regulation is the negative inverse of the fold change. Upregulated genes (fold differences larger than a 2-fold threshold) are indicated in red, downregulated genes (fold differences narrower than 2-fold threshold) are indicated in green. Genes similarly expressed for the Test Group 1 and Control (fold differences between -2 to +2 boundary) are indicated in black. Abbreviations: HLCs, hepatocyte like-cells.

Supplementary Table 6. Fold change (2^(- Delta Delta Ct)) of the normalized gene expression (2^(- Delta Ct)) in the Test Sample (BPF treated Man12 HLCs) divided by the normalized gene expression (2^(- Delta Ct)) in the Control Sample (DMSO treated Man12 HLCs). Fold regulation represents fold change results in a biologically meaningful way. Fold change values greater than one indicate a positive- or an upregulation, and the fold regulation is equal to the fold change. Fold change values less than one indicate a negative or downregulation, and the fold regulation is the negative inverse of the fold change. Upregulated genes (fold differences larger than a 2-fold threshold) are indicated in red, downregulated genes (fold differences narrower than 2-fold threshold) are indicated in green. Genes similarly expressed for the Test Group 1 and Control (fold differences between -2 to +2 boundary) are indicated in black. Abbreviations: HLCs, hepatocyte like-cells.

Supplementary Table 7. Fold change (2^(- Delta Delta Ct)) of the normalized gene expression (2^(- Delta Ct)) in the Test Sample (BPS treated Man12 HLCs) divided by the normalized gene expression (2^(- Delta Ct)) in the Control Sample (DMSO treated Man12 HLCs). Fold regulation represents fold change results in a biologically meaningful way. Fold change values greater than one indicate a positive- or an upregulation, and the fold regulation is equal to the fold change. Fold change values less than one indicate a negative or downregulation, and the fold regulation is the negative inverse of the fold change. Upregulated genes (fold differences larger than a 2-fold threshold) are indicated in red, downregulated

EXCLI Journal 2020;19:1459-1476 – ISSN 1611-2156
Received: September 22, 2020, accepted: October 28, 2020, published: November 04, 2020

genes (fold differences narrower than 2-fold threshold) are indicated in green. Genes similarly expressed for the Test Group 1 and Control (fold differences between -2 to +2 boundary) are indicated in black. Abbreviations: HLCs, hepatocyte like-cells.

Supplementary Table 8. Fold change (2^(- Delta Delta Ct)) of the normalized gene expression (2^(-Delta Ct)) in the Test Sample (BPA treated H9 HLCs) divided by the normalized gene expression (2^(-Delta Ct)) in the Control Sample (DMSO treated H9 HLCs). Fold regulation represents fold change results in a biologically meaningful way. Fold change values greater than one indicate a positive- or an upregulation, and the fold regulation is equal to the fold change. Fold change values less than one indicate a negative or downregulation, and the fold regulation is the negative inverse of the fold change. Upregulated genes (fold differences larger than a 2-fold threshold) are indicated in red, downregulated genes (fold differences narrower than 2-fold threshold) are indicated in green. Genes similarly expressed for the Test Group 1 and Control (fold differences between -2 to +2 boundary) are indicated in black. Abbreviations: HLCs, hepatocyte like-cells.

Supplementary Table 9. Fold change (2^(- Delta Delta Ct)) of the normalized gene expression (2^(-Delta Ct)) in the Test Sample (BPF treated H9 HLCs) divided by the normalized gene expression (2^(-Delta Ct)) in the Control Sample (DMSO treated H9 HLCs). Fold regulation represents fold change results in a biologically meaningful way. Fold change values greater than one indicate a positive- or an upregulation, and the fold regulation is equal to the fold change. Fold change values less than one indicate a negative or downregulation, and the fold regulation is the negative inverse of the fold change. Upregulated genes (fold differences larger than a 2-fold threshold) are indicated in red, downregulated genes (fold differences narrower than 2-fold threshold) are indicated in green. Genes similarly expressed for the Test Group 1 and Control (fold differences between -2 to +2 boundary) are indicated in black. Abbreviations: HLCs, hepatocyte like-cells.

Supplementary Table 10. Fold change (2^(- Delta Delta Ct)) of the normalized gene expression (2^(-Delta Ct)) in the Test Sample (BPS treated H9 HLCs) divided by the normalized gene expression (2^(-Delta Ct)) in the Control Sample (DMSO treated H9 HLCs). Fold regulation represents fold change results in a biologically meaningful way. Fold change values greater than one indicate a positive- or an upregulation, and the fold regulation is equal to the fold change. Fold change values less than one indicate a negative or downregulation, and the fold regulation is the negative inverse of the fold change. Upregulated genes (fold differences larger than a 2-fold threshold) are indicated in red, downregulated genes (fold differences narrower than 2-fold threshold) are indicated in green. Genes similarly expressed for the Test Group 1 and Control (fold differences between -2 to +2 boundary) are indicated in black. Abbreviations: HLCs, hepatocyte like-cells.

Supplementary Table 11. Antibodies employed in immunofluorescence studies.
